# The Stigmatization of Leprosy in India and Its Impact on Future Approaches to Elimination and Control

**DOI:** 10.1371/journal.pntd.0000113

**Published:** 2008-01-30

**Authors:** Jesse T. Jacob, Carlos Franco-Paredes

**Affiliations:** 1 Division of Infectious Diseases, Emory University School of Medicine, Atlanta, Georgia, United States of America; 2 Hospital Infantil de México, Federico Gómez, México City, México; Case Western Reserve University School of Medicine, United States of America

Traditionally, India holds the unenviable position of the origin of leprosy. The disease is thought to have then spread, via trade and war, to China, Egypt, and the Middle East, and later to Europe and the Americas. From antiquity to modernity, Indian society treated leprosy singularly with respect to custom and law, a response shaped by both scientific knowledge and cultural attitudes. India's future challenges in leprosy control include multiple systems of medicine, stigma, and educational knowledge gaps. By looking through the historical window of leprosy in India, we propose that continued success in elimination and control requires a holistic approach addressing these issues (Image 1).

**Figure:**
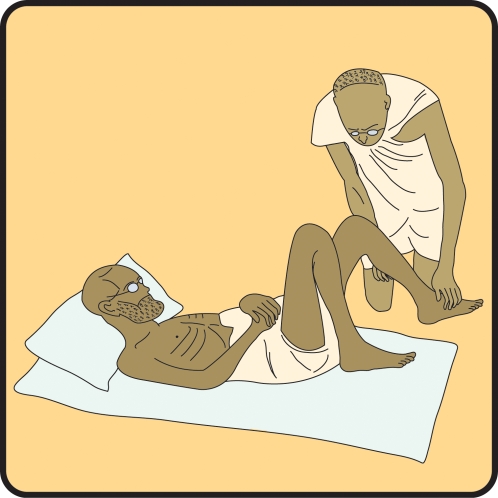


## Leprosy in Ancient India

Early texts, including the *Atharava Veda* (circa 2000 BC) and the *Laws of Manu* (1500 BC), mention various skin diseases translated as leprosy. The Laws prohibited contact with those affected by leprosy and punished those who married into their families, effectively ostracizing those with the disease for their past sins [1]. The *Sushruta Samhita* (600 BC) recommended treating leprosy—or *kushtha*, meaning “eating away” in Sanskrit—with oil derived from the chaulmoogra tree; this remained a mainstay of treatment until the introduction of sulfones [2].

In a legend explaining chalmoogra oil's therapeutic origins, a king banished for his leprosy was instructed to eat the curative seeds of this tree, illustrating the cultural response to leprosy in antiquity: loss of social position and expulsion, even of kings, from the community [3]. Ancient Indian society marginalized those with leprosy because of several factors: its chronic, potentially disfiguring nature; inconsistently effective therapy; association with sin; and the fear of contagion. This combination endowed leprosy with a unique stigma that persists today and resulted in its treatment with both seclusion and medical therapy.

## Leprosy in Colonial India

Soon after their arrival, Europeans described the uncommon practice of ritual suicide by those affected by leprosy, who were often assisted by their families. Though Hinduism generally considers suicide a sin, for leprosy it was not [4]. Christians too associated leprosy with sin. Struck by the scale of this Biblical disease, Europeans, especially missionaries, singled it out from a myriad of tropical infections. They often described the most dramatic forms of disfiguring leprosy, evoking fear of an “imperial danger”: leprosy reaching the British Isles. The public pressured the colonial government for the segregation of people with leprosy.

Three events over a 30-year period strengthened the argument for confinement. First, the first leprosy census in 1872 quantified the problem: over 108,000 cases, for a prevalence of 54 cases/10,000 population. Approximately 1% received organizational support, renewing the cries for segregation to facilitate delivery of care [5]. Next, Hansen identified *Mycobacterium leprae* in 1873 and postulated it as the etiologic, transmissible agent of leprosy. Third, Father Damien, the Belgian missionary priest in Hawaii, contracted leprosy and died in 1889, proving its contagiousness. These events led to the popular consideration of leprosy as a widespread contagious disease requiring containment.

In response, the British government sent its Leprosy Commission (comprising both physicians and administrators) to India to investigate. The commission's report in 1891 concluded that “the amount of contagion which exists is so small that it may be disregarded” [6]. Initially, the colonial government accepted these findings but, under increasing popular pressure from England and within India, enacted the Leprosy Act of 1898. This law institutionalized people with leprosy, using segregation by gender to prevent reproduction. For the self-sufficient individual with leprosy, segregation and medical treatment were voluntary, but vagrants and fugitives from government-designated leprosaria were subject to punitive action. Charities and local governments in British India constructed many new institutions for people with leprosy, providing combined social, religious, and medical services. However, as predicted by the Leprosy Commission, the lack of infrastructure prevented the Leprosy Act from being strictly enforced. It was repealed in 1983 after the advent of effective multi-drug therapy for leprosy.

## Leprosy in Post-Colonial India

Disease control marked the Indian government's initial approach, starting in 1955 with the creation of the National Leprosy Control Program for surveillance. In 1983, with the availability curative multi-drug therapy, the government changed the name to the National Leprosy Elimination Program (NLEP), with a focus on treatment. Starting in 1997, the government conducted several modified leprosy elimination campaigns; these short, concentrated bursts of statewide case detection activities included orientation of all village-level workers and volunteers on leprosy, house-to-house searches in specified areas, and awareness programs using mass media, school activities, and community meetings. State governments also began integrating leprosy care into their general health systems starting in 1997, moving from vertical control programs to horizontal health services, an intervention shown to decrease the stigma associated with leprosy due to family counseling and community outreach [7].

On January 30, 2005 India celebrated the elimination of leprosy as a public health problem after achieving the nationwide prevalence of <1 case/10,000 population, though not without criticism regarding the accuracy and choice of target parameter [8]. This is a remarkable achievement given that in 1981, two years before NLEP, there were nearly 4,000,000 cases with a prevalence of >50 cases/10,000 population [9]. However, in a population of more than a billion people, up to 100,000 people with leprosy remain, representing approximately half of the world's disease burden. Some regions, mostly rural, still have up to five times the national average of cases; these areas have become the next targets in leprosy control [10].

The future of leprosy control and elimination offers several challenges with both structural and cultural dimensions. Efforts to decrease health inequity due to poverty, especially in rural areas with limited access to health care, may help with leprosy control. However, if cultural beliefs are not addressed, increased availability may not translate into an appropriate increase in utilization. Cultural aspects of leprosy affecting its control include traditional medicine and stigma.

Only limited efforts have been made to include the numerous nonallopathic (traditional) practitioners in India in leprosy control and elimination efforts, but their inclusion is important to its success [11]. Indians can seek public or private health care from allopathic (conventional Western) physicians, but often see private practitioners of homeopathy or the three major Indian systems of medicine (ISM) including Ayurveda, Siddha, and Unani. The practitioners of ISMs, who outnumber allopaths in India, continue to use compounded botanicals and agents such as chaulmoogra oil for primary or adjunctive therapy. If this therapy fails, patients are referred to government clinics where free multi-drug allopathic therapy is offered; use of traditional medicine has been shown to be a risk factor for delay in diagnosis [12]. The popularity of ISM can, as least in part, be attributed to two factors: the stigma carried by government-run vertical leprosy clinics and the preference for traditional medicine. Further investigation into the safety and efficacy of ISM therapies is needed, and the possibility of integrating aspects of ISM into the general health system should be evaluated. For example, chalmoogra oil may be effective as adjunctive therapy in wound healing [13]. The effectiveness of leprosy control in this integrated system should be periodically assessed not only in measures of leprosy rates, but of changes in knowledge, attitudes, and practices.

Leprosy continues to be stigmatized in a society with a deeply ingrained, though legally abolished, caste system, partly through lack of knowledge. Socially marginalized groups such as women, “backward classes” (minority social or ethnic groups defined by the government), and the urban poor are less likely to seek care; they often view elimination efforts as problematic because they fail to account for their individual needs [14]. Further, community education and medical knowledge of the disease does not immediately dispel stigma. In one community, only 30% of individuals claiming a high knowledge of leprosy also had a positive attitude toward patients with leprosy [15]. More studies are needed to better understand the causes of stigma and to assess the effect of interventions to decrease it.

Hansen's disease is still called *kusht* in most Indian languages, as it was in Sushrutha's time. The word itself still evokes fear and aversion, despite Mohandas “Mahatma” Gandhi's efforts to destigmatize the disease. Parchure Shastri, a Brahmin and Sanskrit scholar who became an outcast when he acquired leprosy, came to stay in Gandhi's *ashram* in 1939. His contemporaries considered sheltering or touching a person with leprosy unthinkable, but Gandhi changed Shastri's wound dressings and massaged his feet daily. This iconic image (http://commons.wikimedia.org/wiki/Image:Gandhi_leper.jpg) was later depicted on a postage stamp emblazoned with the words “leprosy is curable.” The cultural shift Gandhi desired is materializing; in 2005, representatives of the estimated 630 leprosy colonies in India met in New Delhi. Entitled “Empowerment of People Affected by Leprosy,” this conference sought to demarginalize those affected by the disease and reintegrate them into society.

## Conclusions

The history of leprosy in India offers insights into one of the world's most misunderstood diseases. Furthermore, leprosy control and elimination in India still faces many challenges. Although many of the theoretical and practical approaches of the past have been discarded, their careful examination provides insights for the future. Sustaining the gains made so far and further reducing the disease burden in India require an innovative, holistic approach that includes ongoing education, efforts to identify interventions to dispel stigma, and the inclusion of nonallopathic practitioners in disease control programs.
